# Postauricular congenital alveolar rhabdomyosarcoma- a case report of an unusual entity

**DOI:** 10.1186/1746-1596-1-37

**Published:** 2006-10-17

**Authors:** Mahesha Vankalakunti, Ashim Das, Narasimhan KL Rao

**Affiliations:** 1 Department of Histopathology, Postgraduate Institute of Medical Education & Research, Chandigarh, India; 2 Department of Pediatric surgery, Postgraduate Institute of Medical Education & Research, Chandigarh, India

## Abstract

**Background:**

Congenital alveolar rhabdomyosarcoma is an extremely uncommon and invariably fatal tumor with the current therapy. Less than 25% of patients present with evidence of cutaneous metastasis.

**Case presentation:**

We report a case of congenital alveolar rhabdomyosarcoma in an 18-month-old male who presented with a progressively increasing mass lesion in the left post-auricular region since birth. Radiological examination did not show any intracranial involvement of the mass lesion. Upon resecting the mass that was 10-cm in largest dimension, the gross, microscopic, and ultrastructural findings were consistent with congenital alveolar rhabdomyosarcoma.

**Conclusion:**

The suspicion of alveolar subtype on histological grounds and proper evaluation of this tumor by immunostain and ultrastuctural examination is necessary. In the Medline literature search, there is no report of large congenital alveolar rhabdomyosarcoma in the post-auricle region.

## Background

Although rhabdomyosarcoma (RMS) is an uncommon childhood malignancy, it is the most frequently encountered soft tissue sarcoma found in infants and children [1]. Six percent of all RMS cases occur in children age <1 year at the time of diagnosis [2,3]. RMS is slightly more common in males than females (range, 1.3–1.4: 1). The head and neck, extremities, genitourinary tract, and trunk are the most common primary sites. Overall, < 25% of patients have metastatic spread at the time of diagnosis, with the most common sites including the lung, lymph nodes, bone, and bone marrow.

Congenital alveolar RMS, defined as disease present at birth, is a rare subtype of RMS. We present a case of congenital alveolar RMS, which was completely resected at the age of 18 months.

## Case report

**Figure 1:**
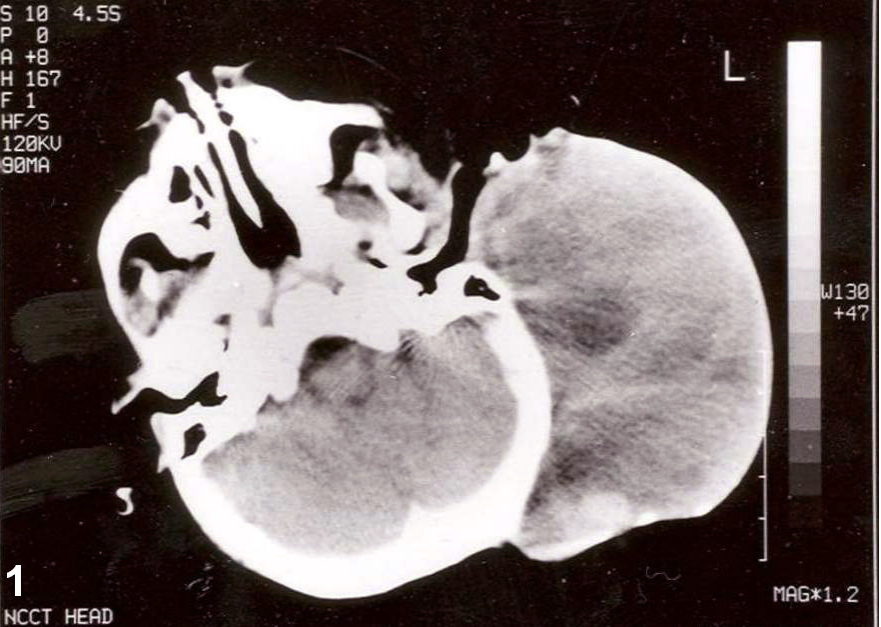
Non-contrast computed tomography of head showing the hypoechoeic mass without any intracranial extension.

An eighteen-month-old male child presented with a mass lesion in the left post-auricle region. The mass was present at birth and was gradually increasing in size. The measurement of the mass was 10 × 6 × 4 cm. The skin over it was unremarkable, and the other systemic examination was within normal limits. A non-contrast computed tomography of head and neck revealed the mass in the left post-auricular region (Figure 1). There was no intracranial involvement.

**Figure 2:**
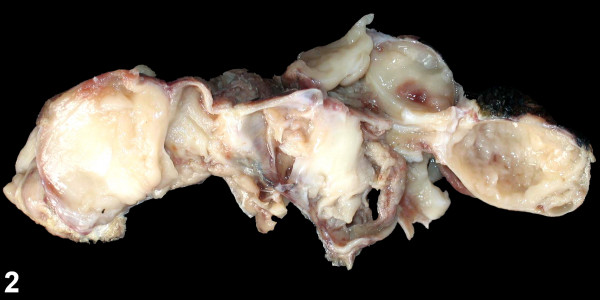
Gross specimen revealing glistening gelatinous gray-white cut surface with cystic areas.

A histologic diagnosis of congenital alveolar rhabdomyosarcoma (RMS) was made after complete excision of the mass. Grossly, the mass was fairly well circumscribed, multinodular with a glistening, gelatinous gray-white surface. Cystic areas were seen in between (Figure 2). Neither haemorrhage nor necrosis was present.

**Figure 3:**
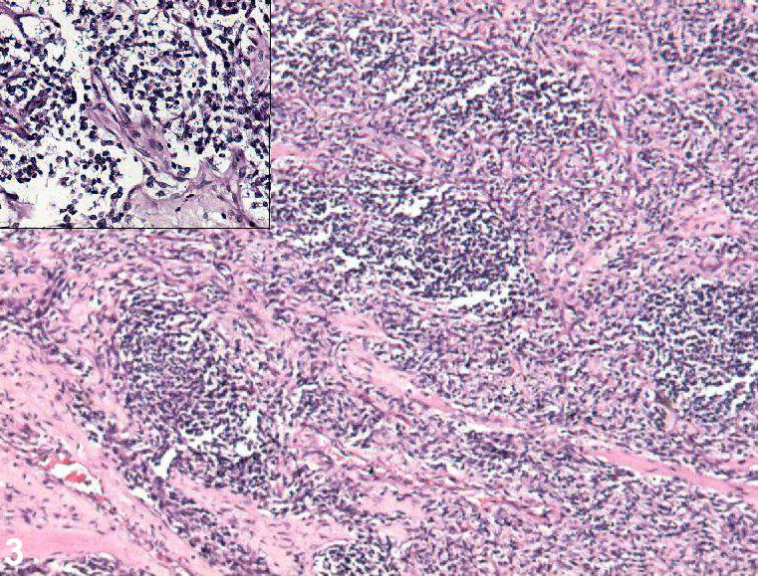
Photomicrograph showing nodules of tumor cells separated by hyalinised fibrous septae (50×, HE stain). *Inset: *Discohesive large tumor cells with hyperchromatic nucleus and scant cytoplasm (200×, HE stain).

**Figure 4:**
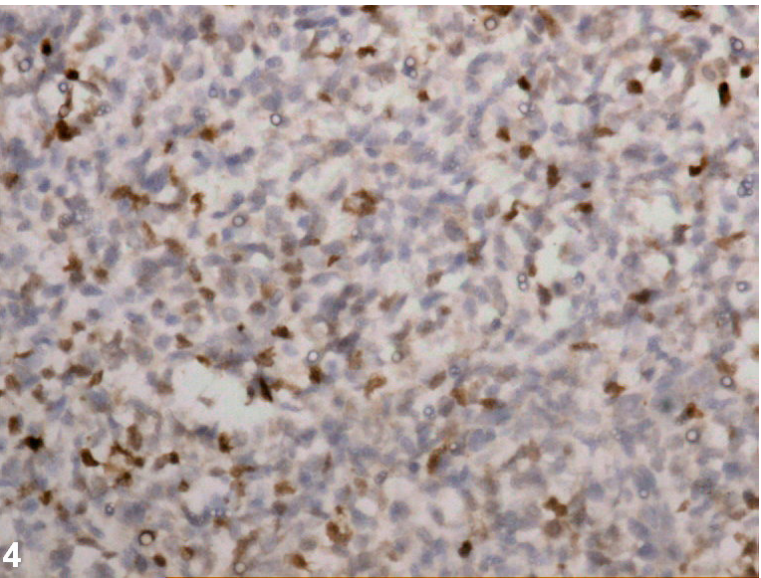
Myogenin immunostain revealing nuclear positivity (200×, immunoperoxidase).

Microscopically, it revealed a varying degree of cellularity with densely packed cellular areas separated by a framework of hyalinized fibrous septa. The tumor cells were discohesive in the centre. The individual cells were large, round with darkly staining hyperchromatic nuclei, inconspicuous nucleoli, and scant indistinct cytoplasm (Figure 3). High mitotic rate was noticed. The tumor cells (≈40%) were positive for desmin and myogenin (Figure 4) immunostains. The other panels of immunostains used were CD45, CD20, MIC-2, CK, Neuron specific enolase, and myeloperoxidase, all of which were negative.

Ultrastructuraly, the tumor cells showed few cytoplasmic organelles and occasional bundles of thin myofilaments measuring 4–6 nm in diameter. Bone marrow examination didn't reveal any metastatic deposit. A cytogenetic analysis was not performed because of technical reasons. In the one year follow-up period, there has been no recurrence or metastatic deposit.

## Discussion

Rhabdomyosarcoma (RMS), a malignant tumor originating from skeletal muscle, usually occurs in the first two decades of life. The vast majority of RMS occurs in head and neck region, extremities, genitourinary system and trunk. RMS bears a close resemblance to various stages in the embryogenesis of normal skeletal muscle, but its pattern is much more variable and ranges from poorly differentiated tumours that are very difficult to diagnose without immunohistochemical or electron microscopic examination to a well-differentiated RMS.

A retrospective study has shown the incidence of tumours in the neonatal period to be 7.2 per 100,000 live births per year (1:13,700) [3]. In the same study, soft tissue tumours represented 17% of all tumours presenting within the first month of life; however, the majority of these tumours (65%) were histologicaly benign [6]. Overall, <1% of all patients with RMS present with the disease within the first month of life and in these cases the histology predominantly is of the embryonal sub-type [3-6]. Alveolar RMS is characterized by specific chromosomal translocations, the majority of which are the t(2;13)(q35;q14) translocation.

Only a few cases (less than 20) have been reported in the literature about alveolar subtype of congenital rhabdomyosarcoma. Alveolar RMS has been previously described in the region of the eyes, ears, infratemporal, cervical and parameningeal regions in neonates and children [7]. But to the best of our knowledge, there has been no record of congenital alveolar RMS in the post-auricle region.

Our case belongs to Group II of clinical staging (Intergroup Rhabdomyosarcoma Studies Classification) [8]. The alveolar type, large size (>5 cm) and location are poor prognostic factors in our case. Morphologically, 'solid' forms of alveolar RMS can also be seen which might simulate the round cell areas of embryonal RMS. However, uniform cellular pattern, multinucleate giant cells and incipient alveolar features, supported by cytogenetic studies help in differentiating between the two. All the morphological features mentioned above were seen in our case. It is important not to confuse the two, as the alveolar type carries a less favourable prognosis.

Differential diagnosis includes neuroblastoma, neuroepithelioma, extra skeletal Ewing's sarcoma, melanotic neuroectodermal tumour of infancy and malignant lymphoma.

The clinical management of neonates with malignancy presents considerable problems due to the physiologic immaturity of many organ systems in infants. Neonates in the Intergroup Rhabdomyosarcoma Studies Classification (IRS) study received 70% of the prescribed dose of the three principal agents used to treat RMS (cyclophosphamide, vincristine, and actinomycin D). Severe myelosuppression and infections were the principal side effects. It is interesting to note that our patient had no recurrence of the lesion or any metastatic deposit elsewhere at the end of one year follow-up. Surgery followed by the standard chemotherapy regimen was given according to the treatment plan. Grundy et al reported the longest survivor who underwent myeloablative therapy with peripheral stem cell support, and hence suggest that more intensive treatment may be of value in this rare condition [9].

## Conclusion

Our case exemplifies the rare sub-type of congenital RMS occurring in the post-auricular area. This is a highly malignant tumor with no record of long-term survivors. In this setting, clinical outcome and radiological findings must be accounted for by both pathologists and clinicians who must be aware of such an unusual presentation of congenital rhabdomyosarcoma, whose diagnosis requires representative tissue sampling and whose treatment may require an adjustment of drug dosage.
